# Non-O blood group is associated with lower risk of in-hospital mortality in non-surgically managed patients with type A aortic dissection

**DOI:** 10.1186/s12872-020-01806-5

**Published:** 2020-12-09

**Authors:** Song Huang, Yequn Chen, Zhaotao Huang, Shiwan Wu, Nianling Xiong, Xiru Huang, Xin Wang, Chang Chen, Bin Wang, Weiping Li, Liangli Hong, Shu Ye, Xuerui Tan

**Affiliations:** 1 The First Affiliated Hospital of Shantou University Medical College, Shantou, 515041 Guangdong China; 2 Shantou University Medical College, Shantou, 515041 Guangdong China; 3 Department of Cardiovascular Sciences and NIHR Leicester Biomedical Research Centre, University of Leicester, Leicester, UK; 4 Clinical Cohort Research Center, The First Affiliated Hospital of Shantou University Medical College, Shantou, China; 5 Clinical Research Center, The First Affiliated Hospital of Shantou University Medical College (SUMC), Shantou, China

**Keywords:** Aortic dissection, Mortality, ABO blood groups

## Abstract

**Background:**

The association between different ABO blood groups and mortality of aortic dissection (AD) remains controversial. This study aimed to examine whether different ABO blood groups affect the prognosis of AD.

**Methods:**

Demographic and clinical data were collected from 877 patients diagnosed with AD from 2015 to 2019 in the First Affiliated Hospital of Shantou University Medical College. The association between in-hospital mortality of AD patients and ABO blood group was analyzed using Cox proportional hazards regression models.

**Results:**

This retrograde cohort study demonstrated that for 877 patients, male gender, non-O blood group, Stanford type B AD (TBAD), higher presenting systolic and diastolic blood pressure, and being a recipient of aortic arch replacement surgery (surgery) or endovascular stent-graft implantation (stent-graft) were associated with decreased in-hospital mortality of AD. In Cox proportional hazards models, non-O blood group was associated with lower risk of early mortality regardless of adjustment (HR = 0.668, 95% confidence interval [CI] 0.473–0.944 before adjustment, HR = 0.662, 95% CI 0.468–0.935 after adjustment for age and sex, and HR = 0.641, 95% CI 0.453–0.906 after adjustment for AD types, SBP and surgery). Further analyses revealed that for patients diagnosed with type A AD (TAAD), non-O blood group renders a significant 34.3% decrease in the risk of in-hospital mortality compared with blood group O. Specifically, this difference in mortality risk was found among TAAD patients who did not undergo surgery (HR = 0.579, 95% CI 0.377–0.889), rather than those who did. There was no significant difference in early mortality for patients with TBAD, whether or not stent-grafts were implanted.

**Conclusions:**

Non-O blood type decreases the risk of in-hospital mortality, especially for TAAD, in AD patients without surgical intervention. More attention must be paid to blood type O TAAD patients without surgical interventions, and early surgical intervention may be an effective means to decrease in-hospital mortality of TAAD.

## Background

Aortic dissection (AD) is a life-threatening emergency with an overall mortality of 27.4% [1]. It is well established that the outcome of AD is associated with multiple factors, such as age, gender, type of AD, aortic diameter and presenting blood pressure [2–4]. It has also been frequently proposed that individual ABO blood groups affect serum cholesterol concentration, inflammation and hemostatic status [5–7], all of which could independently alter the course of AD [8–10]. Furthermore, previous studies have associated ABO blood group with other cardiovascular diseases such as atherosclerosis and coronary heart disease [11], the former being a risk factor for and contributor to the pathogenesis of AD [12]. Nonetheless, the relationship between ABO blood group and AD prognosis has attracted little focus. In this study, we investigate the association of ABO blood group and the in-hospital mortality of AD.

## Methods

### Study population

**Fig. 1:**
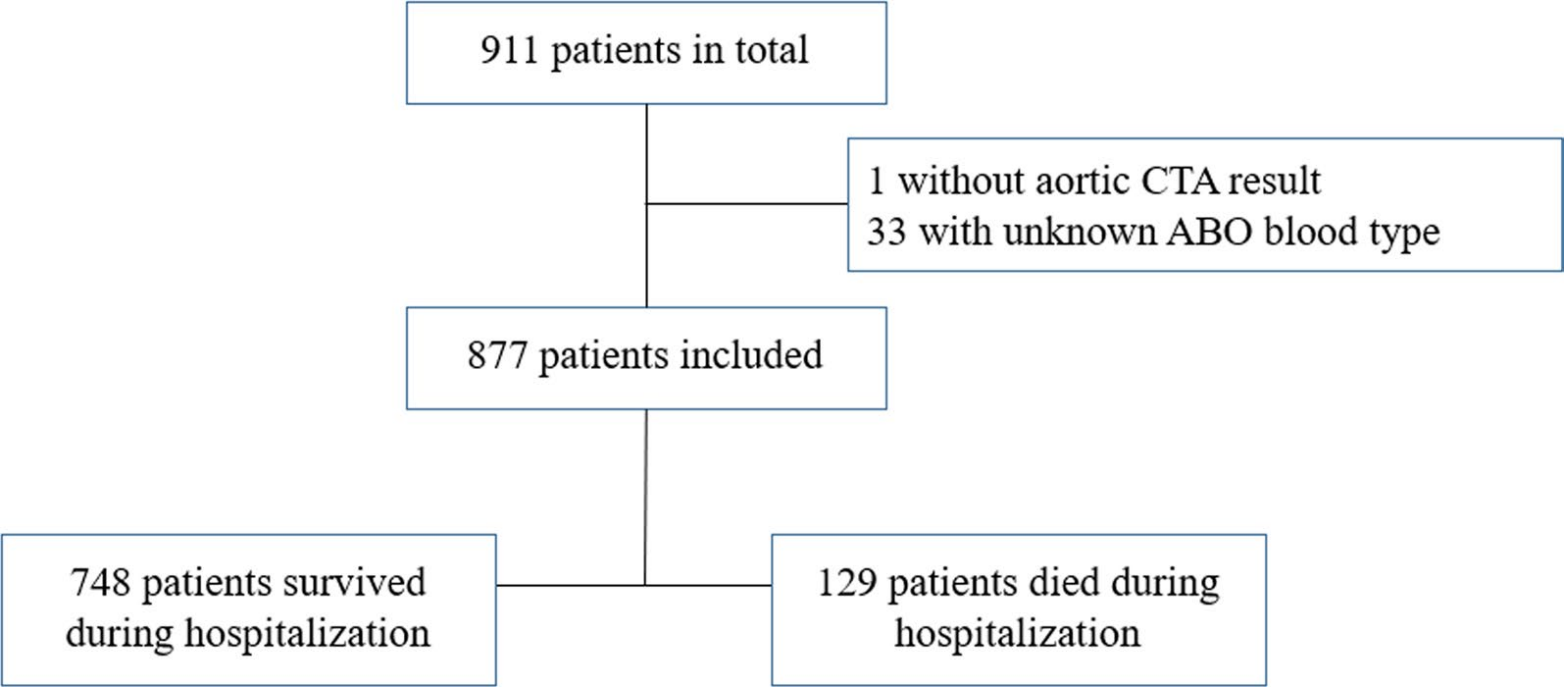
Flowchart of patient enrollment. *AD* aortic dissection, *CTA* computed tomographic angiography

This retrospective cohort study involved 911 recruited Chinese patients diagnosed with AD from January 2015 to July 2019 in the First Affiliated Hospital of Shantou University Medical College in Shantou, China. Computed tomographic angiography of the aorta was performed for confirmatory diagnosis of AD. The Stanford classification was applied to determine the types of AD. In this classification system, type A aortic dissection (TAAD) is defined as an intimal tear involving the ascending aorta whereas type B aortic dissection (TBAD) does not [13]. Demographic and clinical data including age, gender, ABO blood group, systolic blood pressure (SBP), diastolic blood pressure (DBP), type of AD, presence of aortic arch replacement surgery (surgery) or endovascular stent-graft implantation (stent-graft), history of hypertension or diabetes mellitus and causes of mortality were collected from patient hospital records. Thirty-four patients with incomplete data were excluded (Fig. 1). This study was conducted anonymously with regard to data collection and analysis, and was approved by the Research Ethics Committee of the First Affiliated Hospital of Shantou University Medical College. Informed consent was waived due to the retrospective nature of the study.

### Statistical analysis

Quantitative data distribution was assessed by a Kolmogorov–Smirnov test of normality. Patients were classified based on their in-hospital outcome into surviving and non-surviving groups. For comparisons of patient baseline characteristics between the two groups, Mann–Whitney U tests were used for continuous variables and chi-square tests for categorical variables. To determine the hazard ratio and corresponding 95% confidence interval (CI) for the association between blood type and mortality rate of AD, the Cox proportional hazards model was used with the duration of hospitalization being the time scale. We established three models which were adjusted for confounders to examine if ABO blood group served as an independent predictor of all-cause mortality and mortality caused by aortic dissection rupture (ADR). Model 1 was unadjusted; Model 2 was adjusted for age and sex; and Model 3 was adjusted for clinical characteristics determined by the following method: (1) using univariate Cox proportional hazards models, potential clinical characteristics with a *p* value of less than 0.10 were included for the next step, then (2) a multivariate step-wise logistic regression model was performed to further select the meaningful clinical characteristics that were then incorporated in Model 3. Furthermore, considering the potential interactions among the AD types, surgical management and ABO blood group [7] and their overall influences on AD mortality, we divided our patients into two groups based on their type of AD (TAAD or TBAD). Subsequently, we separated them into surgery and non-surgery subgroups within the TAAD group, and stent-graft and non-stent-graft subgroups within the TBAD group. Within these groups and subgroups, we then analyzed the association between ABO blood groups and in-hospital mortality (all-cause and ADR-related mortality respectively). An additional model was used to compare individual blood groups (A, B, AB) with blood group O in terms of early mortality. All statistical analyses were performed with SPSS version 20.0 (SPSS Inc., Chicago, Illinois, USA). A *p* value of less than 0.05 was considered statistically significant.

## Results

Baseline characteristics of the study population are presented in Table 1 and quantitative data (age, SBP and DBP) normality are presented in Additional file 1: Table S1. Eight hundred seventy-seven AD patients were included from January 2015 to July 2019. Male gender, non-O blood group, TBAD, higher SBP or DBP, and receipt of surgery or stent-grafting were associated with significantly lower in-hospital mortality. The most common cause of in-hospital death was ADR, followed by arrhythmia and septic shock (Additional file 1: Table S2).

**Table 1:** Baseline characteristics of study participants

Variable	Surviving	Non-surviving	*p* value
	(n = 748)	(n = 129)	
Age	62 (52–69)	64 (53–72)	0.099
Male gender	595 (79.5)	89 (69.0)	0.008
Blood type O	311 (41.6)	68 (52.7)	0.018
TAAD	282 (37.7)	100 (77.5)	0.000
SBP	158 (137–179)	136 (113–161)	0.000
DBP	91 (78–104)	80 (62–92)	0.000
Receipt of surgery	154 (20.6)	15 (11.6)	0.017
Stent-graft implantation	144 (19.3)	6 (4.7)	0.000
Hypertension	656 (87.7)	108 (83.7)	0.213
Diabetes	72 (9.6)	8 (6.2)	0.212

A Kolmogorov–Smirnov test of normality was performed for all quantitative values. The quantitative values (age, SBP, DBP) were not normally distributed. Data are presented as the median (interquartile range) or number (percentage). values of continuous and categorical variables were obtained by the Mann–Whitney U test or χ^2^ test, respectively. Surgery: aortic arch replacement surgery. Stent-graft: aortic stent-graft implantation

*TAAD* Stanford type A aortic dissection, *SBP* systolic blood pressure, *DBP* diastolic blood pressure

**Fig. 2:**
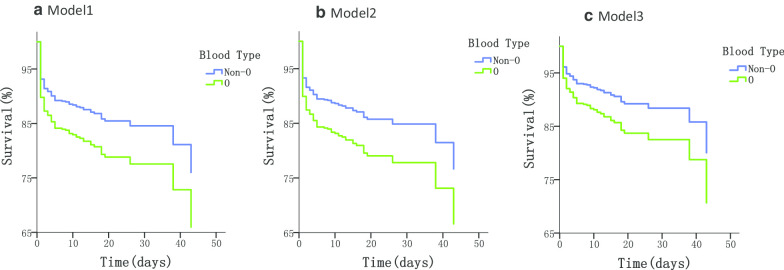
Survival curves of O-type and Non-O-type AD patients. Survival curves were obtained using Cox proportional hazards models. **a** Model 1 was unadjusted; **b** Model 2 was adjusted for age, sex; **c** Model 3 was adjusted for AD type, SBP and surgery. *AD Type* aortic dissection type, *SBP* systolic blood pressure

After being selected by the combination of univariate Cox proportional hazards models and a multivariate stepwise logistic regression model, non-O blood group, TBAD, higher SBP and surgical intervention remained associated with decreased in-hospital all-cause mortality (Additional file 1: Tables S3, S4). In the unadjusted Cox proportional hazards model, non-O blood group presented a significant 33.2% decrease in the risk of in-hospital mortality compared with blood group O (HR = 0.668, 95% CI 0.473–0.944). In Model 2 (adjusted for age and sex) and Model 3 (adjusted for AD type, SBP and surgery), the protective effect was still observed (HR = 0.662, 95% CI 0.468–0.935 and HR = 0.641 95% CI 0.453–0.906 respectively) (Table 2, Fig. 2). The same method was applied in analyzing the in-hospital mortality caused by ADR and showed that the non-O blood group was significantly associated with lower risk of ARD-related death irrespective of adjustment (Additional file 1: Tables S5–7).

**Table 2:** Associations of blood type with risk of in-hospital mortality in AD patients

Independent variable	Model 1	Model 2	Model 3
	HR (95% CI)	HR (95% CI)	HR (95% CI)
Non-O blood group	0.668 (0.473–0.944)*	0.662 (0.468–0.935)*	0.641 (0.453–0.906)*
Age		1.010 (0.995–1.024)^&^	
Female gender		1.580 (1.083–2.304)*	
TBAD			0.138 (0.090–0.218)*
SBP			0.987 (0.981–0.992)*
Receipt of surgery			0.129 (0.073–0.227)*

Values are based on Cox proportional hazards models. Results are shown as the hazard ratio (95% confidence interval), and statistical significance is indicated when the 95% CI does not contain the value 1. In the model, O-type was set as the reference. Model 1 was unadjusted; Model 2 was adjusted for age and sex; Model 3 was adjusted for AD type, SBP and surgery. The co-variates incorporated in Model 3 were selected based on the results of univariate Cox proportional hazards models and a multivariate step-wise logistic regression model

*AD* aortic dissection, *TBAD* type B aortic dissection, *SBP* systolic blood pressure, *HR* hazard ratio, *95% CI* 95% confidence interval

^*^p value < 0.05

^&^*p* value > 0.05

**Fig. 3:**
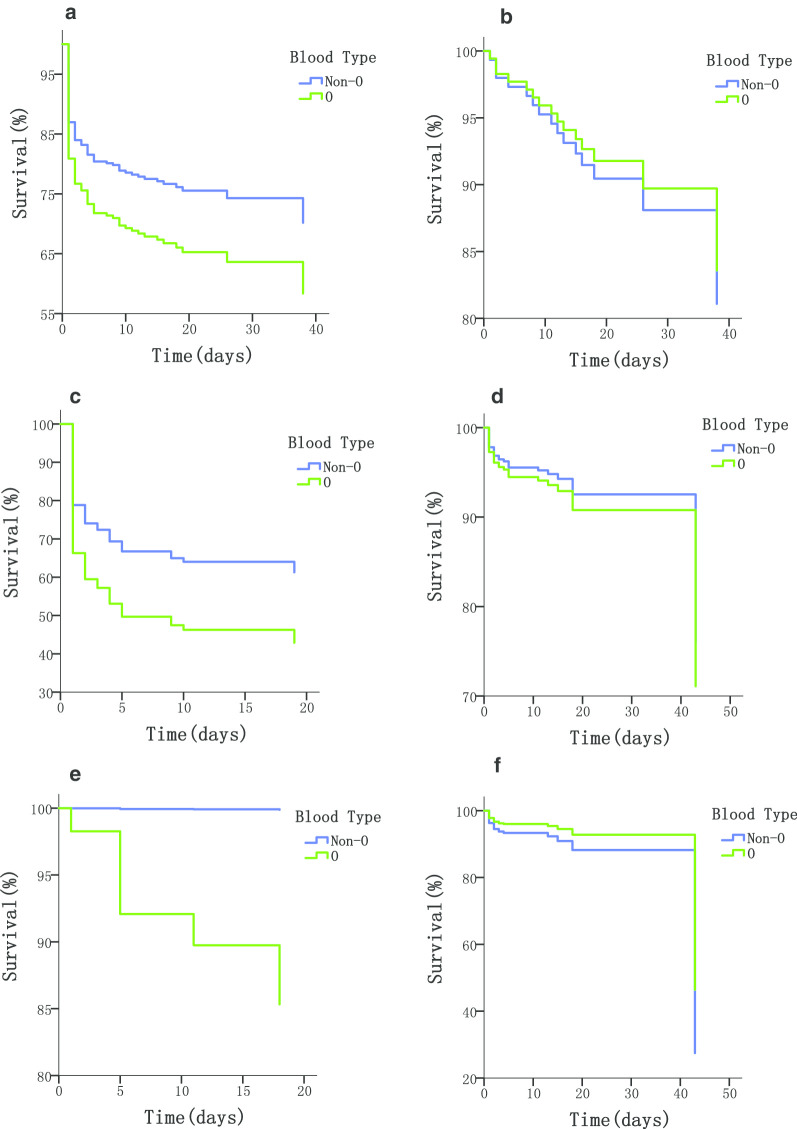
Survival curves of patient subgroups. Survival curves were obtained using Cox proportional hazards models. Survival curve for the O-type and non-O-type patients in subgroup **a** TAAD, **b** TAAD and surgery, **c** TAAD and non-surgery, **d** TBAD, **e** TBAD and stent-graft, and **f** TBAD and non-stent-graft. *TAAD* Stanford type A aortic dissection, *TBAD* Stanford type B aortic dissection

For further analysis, patients were divided into 2 groups based on their type of AD (Tables 3, 4, Fig. 3). In the TAAD group, risk of in-hospital mortality in patients with non-O blood type was 34.3% lower than those with blood type O (HR = 0.657, 95% CI 0.440–0.975). After dividing these patients into surgery and non-surgery subgroups, the non-O blood groups remained associated with lower risk of early mortality (HR = 0.579, 95% CI 0.377–0.889), notably caused by ADR (Additional file 1: Tables S8, S9), in TAAD patients without surgery. However, in the TAAD surgery subgroup, different blood types presented no significant difference in in-hospital mortality risk (HR = 1.169, 95% CI 0.414–3.305). In the TBAD group, no association was found between ABO blood group and mortality of AD, regardless of the involvement of a stent-graft procedure.

**Table 3:** Associations of blood type with in-hospital mortality in TAAD patients

Subgroup	Blood type	Surviving	Non-surviving	HR (95% CI)
TAAD	Non-O	168 (59.6%)	46 (46%)	0.657 (0.44–0.975)
	O	114 (40.4%)	54 (54%)	
TAAD, Surgery	Non-O	81 (55.5%)	9 (60.0%)	1.169 (0.414–3.305)
	O	65 (44.5%)	6 (40.0%)	
TAAD, Non-surgery	Non-O	87 (64.0%)	37 (43.5%)	0.579 (0.377–0.889)
	O	49 (36.0%)	48 (56.5%)	

Values are based on Cox proportional hazards models. Data are presented as number (percentage). Results are shown as the hazard ratio (95% confidence interval). In the models, O-type was set as the reference

*TAAD* Stanford type A aortic dissection, *HR* hazard ratio, *95% CI* 95% confidence interval

**Table 4:** Associations of blood type with in-hospital mortality in TBAD patients

Subgroup	Blood type	Surviving	Non-surviving	HR (95% CI)
TBAD	Non-O	269 (57.7%)	15 (51.7%)	0.803 (0.387–1.666)
	O	197 (42.3%)	14 (48.3%)	
TBAD, Stent-graft	Non-O	81 (61.4%)	0	0.008 (0.000–8.813)
	O	51 (38.6%)	6 (100%)	
TBAD, Non-stent-graft	Non-O	188 (56.3%)	15 (65.2%)	1.684 (0.686–4.134)
	O	146 (43.7%)	8 (34.8%)	

Values are based on Cox proportional hazards models. Data are presented as number (percentage). Results are shown as the hazard ratio (95% confidence interval). In the models, O-type was set as the reference

*TBAD* Stanford type B aortic dissection, *HR* hazard ratio, *95% CI* 95% confidence interval

**Fig. 4:**
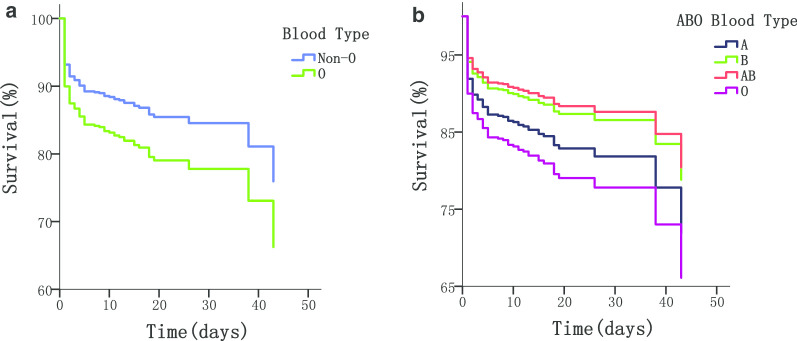
ABO blood type-dependent survival of AD patients. **a** O-type vs non-O-type; **b** O-type vs non-O-type (AB, A, or B)

Interestingly, when analyzing individual ABO blood groups, patients with blood type AB seemed to have the best prognosis (Fig. 4), followed closely by blood group B, A, and then O. However, statistically significant lower mortality was only observed in blood type B as compared with blood type O (HR = 0.575, 95% CI 0.361–0.916) (Table 5). The difference in mortality was neither significant between blood type AB and O (HR = 0.526, 95% CI 0.212–1.306), nor between blood type A and O (HR = 0.799, 95% CI 0.525–1.216).

**Table 5:** Association of ABO blood type with in-hospital mortality in AD patients

Blood Type	Surviving	Non-surviving	HR (95% CI)
	n = 748	n = 129	
AB	47 (6.3%)	5 (3.9%)	0.526 (0.212–1.306)
A	187 (25%)	32 (24.8%)	0.799 (0.525–1.216)
B	203 (27.1%)	24 (18.6%)	0.575 (0.361–0.916)
O	311 (41.6%)	68 (52.7%)	
Non-O	437 (58.4%)	61 (47.3%)	0.668 (0.473–0.944)
O	311 (41.6%)	68 (52.7%)	

Data are presented as number (percentage). Results are expressed as the hazard ratio (95% confidence interval) derived from Cox proportional hazards model. In the model, O-type was set as the reference

*AD* aortic dissection, *HR* hazards ratio

## Discussion

The main finding of this study is that patients with a non-O blood type have considerably lower risk of early mortality than those with blood type O. In particular, this trend is exhibited by patients who were diagnosed with TAAD but did not receive standard surgical intervention. Despite the advances in surgical skills and improved prognosis of AD patients over the past two decades [14], AD remains a medical catastrophe, making it essential to identify patients who have a lower chance of survival and require additional attention. Previous studies have proposed several variables, including age, gender and certain medical conditions, as predictors of AD prognosis [15–19]. In this study, we find male gender, TBAD, higher presenting SBP and DBP and surgical intervention are related to better outcome, basically in line with the results of previous research. In addition, we sought to establish a relationship between AD mortality and ABO blood group, the latter a readily accessible and yet potentially important clinical feature. To be noted, this relationship has also been investigated in two other studies. Nikola et al. designed a case–control study involving 115 patients with type III AD and revealed no significant difference in mortality between various blood groups [20]. A more recent study concerning the surgical prognosis of AD, by Nozohoor et al., included a larger population from The Nordic Consortium for Acute Type A Aortic Dissection database [21]. The authors also found no association between individual ABO blood types and the surgical outcome of AD, except that blood group A was related to poorer long-term prognosis. Although patients with blood type O have a lower level of serum von Willebrand factor (vWF) [22], and hence a stronger tendency to bleed during and after surgery, the authors suggested that the impact of other surgical complications more frequently seen in non-O blood group, such as thromboembolic events [23], outweigh that of hemorrhaging in the long run. While Nozohoor’s study is in many ways comparable to ours, the former was a multi-center study and the latter was a single-center one. More importantly, Nozohoor and colleagues were more concerned with the postoperative outcome of the subjects, whereas a major portion of our patients did not receive surgery or stent-grafts. It is in these non-surgically managed patients that we observed the opposite trend, with non-O blood group being related to lower in-hospital mortality of TAAD. Furthermore, we found non-O blood group also significantly decreases the risk of mortality caused by ADR, the major cause of death for AD patients [24, 25], in TAAD patients without surgery. However, how ABO blood group is related to AD mortality or ADR has not been investigated, and more research is needed to elucidate the mechanism.

Our findings in the non-surgery subgroup also reflect the deeper goal of the study. Although implicated, some of our patients failed to receive surgical intervention due to realistic issues, such as patient preference and their financial status, and it is of equal importance to note that many patients died before surgery was available, due in a large part to lack of sufficient blood stored for transfusion. This finding should drive management decision-making. As treatment strategies are individualized based on age, comorbidities, and factors that might discourage surgery, physicians should take into account other factors that could render their patients at increased risk of death without operation, such as ABO blood group in this case. Furthermore, the significance of blood unit preparation needs to be addressed. Since blood type O is associated with a higher early mortality rate, possibly due to risk of bleeding complications, sufficient storage and supply of O-type blood is essential, especially in areas populated by a higher percentage of people with blood type O, such as South America [26].

Limitations of the study need to be acknowledged. First, this is a single-center study, and our subjects may not be representative of all AD patients. In addition, we did not study how ABO blood group could affect the outcome of AD, and further research can be designed to investigate the molecular mechanism of this process.

## Conclusion

To summarize, non-O blood group is associated with lower risk of in-hospital mortality for patients with TAAD who do not receive surgical intervention. More attention must be paid to blood type O TAAD patients for whom early surgical intervention and adequate supply of O-type blood should be considered. More research is needed to better understand the mechanism of relationship between blood groups and AD.

## Supplementary Information

**Additional file 1: Table S1**. Kolmogorov-Smirnov test of normality. **Table S2**. Distribution of various death causes in O and non-O blood groups. **Table S3**. Univariate Cox proportional hazards analyses of all-cause mortality. **Table S4**. Multivariate stepwise logistic regression analyses of all-cause mortality. **Table S5**. Univariate Cox proportional hazards analyses of ADR-related mortality. **Table S6**. Multivariate stepwise logistic regression analyses of ADR-related mortality. **Table S7**. Associations of blood type with risk of in-hospital mortality caused by ADR. **Table S8**. Associations of blood type with causes of in-hospital mortality in TAAD patients. **Table S9**. Associations of blood type with causes of in-hospital mortality in TBAD patients.

## Data Availability

The datasets used and analyzed during the current study are available from the corresponding author on reasonable request.

## Abbreviations

AD: Aortic dissection
TAAD: Stanford type A AD
TBAD: Stanford type B AD
CI: Confidence interval
SBP: Systolic blood pressure
DBP: Diastolic blood pressure
ADR: Aortic dissection rupture
