# Supplementary material for: Non-O blood group is associated with lower risk of in-hospital mortality in non-surgically managed patients with type A aortic dissection
Source: BMC Cardiovasc Disord. 2020 Dec 9;20:515. doi: 10.1186/s12872-020-01806-5 (PMC7727136; doi:10.1186/s12872-020-01806-5)
Supplement: Supplementary file 1 — Additional file 1: Table S1. Kolmogorov-Smirnov test of normality. Table S2. Distribution of various death causes in O and non-O blood groups. Table S3. Univariate Cox proportional hazards analyses of all-cause mortality. Table S4. Multivariate stepwise logistic regression analyses of all-cause mortality. Table S5. Univariate Cox proportional hazards analyses of ADR-related mortality. Table S6. Multivariate stepwise logistic regression analyses of ADR-related mortality. Table S7. Associations of blood type with risk of in-hospital mortality caused by ADR. Table S8. Associations of blood type with causes of in-hospital mortality in TAAD patients. Table S9. Associations of blood type with causes of in-hospital mortality in TBAD patients. [file 12872_2020_1806_MOESM1_ESM.doc]

**Supplementary Table S1****. Kolmogorov-Smirnov test of normality**

| **Variable** | ***P*-Value** |
| --- | --- |
| Age | 0.000 |

| SBP | 0.003 |
| --- | --- |
| DBP | 0.005 |

*P*-value＜0.05 indicates that the quantitative values(age, SBP, DBP) are not normally distributed.

Abbreviations: SBP, systolic blood pressure; DBP, diastolic blood pressure.

**Supplementary Table S2. Distribution of various death causes in O and non-O blood groups**

| **Cause of death** | **Non-O blood group (n=498)** | **Blood group O (n=379)** | ***P*-Value** |
| --- | --- | --- | --- |
| Arrhythmia (%) | 9 (1.8) | 16 (4.2) | 0.033 |
| ADR (%) | 31 (6.2) | 39 (10.3) | 0.028 |
| Septic shock (%) | 12 (2.4) | 4 (1.1) | 0.138 |
| Others (%) | 9 (1.8) | 9 (2.4) | 0.557 |

Data are presented as number (percentage).

Abbreviations: ADR: aortic dissection rupture.

**Supplementary Table S3. Univariate Cox proportional hazards analyses of all-cause mortality**

| **Variable** | **HR (95%CI)** | ***P*-Value** |
| --- | --- | --- |
| Age | 1.012 (0.998-1.026) | 0.101 |
| Male gender | 1.626 (1.119-2.361) | 0.011 |
| Blood type O | 1.497 (1.059-2.115) | 0.022 |
| TBAD | 0.215 (0.142-0.325) | 0.000 |
| SBP | 0.984 (0.979-0.989) | 0.000 |
| DBP | 0.974 (0.965-0.982) | 0.000 |
| Receipt of surgery | 0.408 (0.236-0.704) | 0.001 |
| Stent-graft | 0.196 (0.086-0.445) | 0.000 |
| Hypertension | 0.722 (0.452-1.153) | 0.172 |
| Diabetes | 0.596 (0.291-1.219) | 0.156 |

Values are based on univariate Cox proportional hazards models. Results are shown as hazard ratio (95% confidence interval) and *p*-value.

Surgery: aortic arch replacement.

Stent-graft: aortic stent-graft implantation

Abbreviations: TBAD, Stanford type B aortic dissection; SBP, systolic blood pressure; DBP, diastolic blood pressure.

**Supplementary Table S****4. Multivariate stepwise logistic regression analyses of all-cause mortality**

| **Variable** | **OR (95%CI)** | ***P*-Value** |
| --- | --- | --- |
| Non-O blood group | 0.583 (0.384-0.885) | 0.011 |
| TBAD | 0.120 (0.075-0.192) | 0.000 |
| SBP | 1.015 (1.009-1.022) | 0.000 |
| Receipt of surgery | 0.155 (0.084-0.285) | 0.000 |

Values are based on the multivariate stepwise logistic regression model, which was adjusted for gender, blood type, AD type, SBP, DBP, surgery and stent-graft. In this model, blood type, AD type, SBP and surgery were inserted step-wise. Results are shown as odds ratio (95% confidence interval) and *p*-value.

Surgery: aortic arch replacement.

Abbreviations: AD, aortic dissection; TBAD, Stanford type B aortic dissection; SBP, systolic blood pressure.

**Supplementary Table S5: Univariate Cox proportional hazards analyses of ADR-related mortality**

| **Variable** | **HR (95%CI)** | ***P*-Value** |
| --- | --- | --- |
| Age | 1.015 (0.995-1.034) | 0.143 |
| Gender | 1.658 (1.001-2.746) | 0.050 |
| Blood type O | 1.671 (1.043-2.678) | 0.033 |
| TBAD | 0.193 (0.109-0.341) | 0.000 |
| SBP | 0.980 (0.974-0.987) | 0.000 |
| DBP | 0.968 (0.957-0.980) | 0.000 |
| Receipt of surgery | 0.217 (0.079-0.596) | 0.003 |
| Stent-graft | 0.190 (0.060-0.605) | 0.005 |
| Hypertension | 0.557 (0.310-1.001) | 0.050 |
| Diabetes | 0.567 (0.207-1.555) | 0.270 |

Values are based on univariate Cox proportional hazards models. Results are shown as hazard ratio (95% confidence interval) and *p*-value.

Surgery: aortic arch replacement.

Stent-graft: aortic stent-graft implantation

Abbreviations: ADR, aortic dissection rupture; TBAD, Stanford type B aortic dissection; SBP, systolic blood pressure; DBP, [diastolic](link:diastolic) [blood](link:blood) [pressure](link:pressure).

**Supplementary Table S6: Multivariate stepwise logistic regression analyses of ADR-related mortality**

| **Variable** | **OR (95%CI)** | ***P*-Value** |
| --- | --- | --- |
| Non-O blood type | 0.537(0.315-0.915) | 0.022 |
| TBAD | 0.141(0.075-0.264) | 0.000 |
| SBP | 0.987(0.975-1.000) | 0.050 |
| Receipt of surgery | 0.081(0.028-0.231) | 0.000 |

Values are based on the multivariate stepwise logistic regression model, which was adjusted for gender, blood type, AD type, SBP, DBP, surgery, stent-graft and hypertension. In this model, blood type, AD type, SBP and surgery were inserted step-wise. Results are shown as odds ratio (95% confidence interval) and *p*-value.

Surgery: aortic arch replacement.

Abbreviations: ADR, aortic dissection rupture; TBAD, Stanford type B aortic dissection; SBP, systolic blood pressure.

**Supplementary Table S7. Associations of blood type with risk of in-hospital mortality caused by ADR**

| Independent variable | Model 1 | Model 2 | Model 3 |
| --- | --- | --- | --- |
|  | HR (95%CI) | HR (95%CI) | HR (95%CI) |
| Non-O blood group | 0.598 (0.373-0.959)* | 0.590 (0.368-0.946)* | 0.588 (0.366-0.942) * |
| Age |  | 1.013 (0.993-1.033) ^&^ |  |
| Female gender |  | 1.600 (0.961-2.666) ^&^ |  |
| TBAD |  |  | 0.129 (0.072-0.234) * |
| SBP |  |  | 0.983 (0.976-0.991) * |
| Recipient of surgery |  |  | 0.068 (0.024-0.192) * |

Values are based on Cox proportional hazards models. Results are shown as the hazard ratio (95% confidence interval), and statistical significance is indicated when the 95% CI does not contain the value 1. In the models, O type was set as the reference.

Model 1 was unadjusted; Model 2 was adjusted for age and gender; Model 3 was adjusted for AD type, SBP and surgery. The co-variates incorporated in Model 3 were selected based on the results of univariate Cox proportional hazards models and a multivariate step-wise logistic regression model.

Abbreviations: ADR, aortic dissection rupture; AD, aortic dissection; TBAD, type B aortic dissection; SBP, systolic blood pressure; HR, hazard ratio; 95% CI: 95% confidence interval.

**P*-value＜0.05

^&^*P*-value＞0.05

**Supplementary Table S8.** **Associations of blood type with causes of in-hospital mortality in** **TAAD patients**

| **Subgroup** | **Cause of death** | **Non-O blood group** | **Blood group O** | **HR (95% CI)** |
| --- | --- | --- | --- | --- |
| TAAD | Arrhythmia | 8 (3.7) | 14 (8.3) | 0.445 (0.187-1.061) |
|  | ADR | 23 (10.7) | 32 (19.0) | 0.559 (0.327-0.955) |
|  | Septic shock | 10 (4.7) | 3 (1.8) | 2.455 (0.673-8.956) |
|  | Others | 5 (2.3) | 5 (3.0) | 0.782 (0.226-2.703) |
| TAAD, Surgery | Arrhythmia | 1 (1.1) | 1 (1.4) | 0.821 (0.051-13.123) |
|  | ADR | 2 (2.2) | 2 (2.8) | 0.814 (0.115-5.776) |
|  | Septic shock | 6 (6.7) | 3 (4.2) | 1.519 (0.376-6.142) |
|  | Others | 0 (0) | 0 (0) |  |
| TAAD, Non-surgery | Arrhythmia | 7 (5.6) | 13 (13.4) | 0.408 (0.163-1.022) |
|  | ADR | 21 (16.9) | 30 (30.9) | 0.527 (0.302-0.920) |
|  | Septic shock | 4 (3.2) | 0 (0) | 48.005 (0.012-190764.973) |
|  | Others | 5 (4.0) | 5 (5.2) | 0.759 (0.220-2.623) |

Values are based on Cox proportional hazards models. Data are presented as number (percentage). Results are shown as the hazard ratio (95% confidence interval).

In the models, O type was set as the reference.

Abbreviations: TAAD, Stanford type A aortic dissection; HR, hazard ratio; 95% CI: 95% confidence interval; ADR: aortic dissection rupture.

**Supplementary Table S9. Associations of blood type with causes of in-hospital mortality in TBAD patients**

| **Subgroup** | **Cause of death** | **Non-O blood group** | **Blood group O** | **HR (95% CI)** |
| --- | --- | --- | --- | --- |
| TBAD | Arrhythmia | 1 (0.4) | 2 (0.9) | 0.351 (0.032-3.878) |
|  | ADR | 8 (2.8) | 7 (3.3) | 0.846 (0.307-2.333) |
|  | Septic shock | 2 (0.7) | 1 (0.5) | 1.464 (0.133-16.142) |
|  | Others | 4 (1.4) | 4 (1.9) | 0.792 (0.196-3.201) |
| TBAD, Stent-graft | Arrhythmia | 0 (0) | 2 (3.5) | 0.007 (0.000-1726.316) |
|  | ADR | 0 (0) | 3 (5.3) | 0.008 (0.000-1555.407) |
|  | Septic shock | 0 (0) | 0 (0) |  |
|  | Others | 0 (0) | 1 (1.8) | 0.009 (0.000-1317.677) |
| TBAD, Non-stent-graft | Arrhythmia | 1 (0.5) | 0 (0) | 50.984 (0.000-615667280.3) |
|  | ADR | 8 (3.9) | 4 (2.6) | 1.524 (0.459-5.060) |
|  | Septic shock | 2 (1.0) | 1 (0.6) | 1.639 (0.148-18.133) |
|  | Others | 4 (2.0) | 3 (1.9) | 1.645 (0.300-9.025) |

Values are based on Cox proportional hazards models. Data are presented as number (percentage). Results are shown as the hazard ratio (95% confidence interval).

In the models, O type was set as the reference.

Abbreviations: TBAD, Stanford type B aortic dissection; HR, hazard ratio; 95% CI: 95% confidence interval; ADR: aortic dissection rupture.
